# The risk and prognostic factors for brain metastases in esophageal cancer patients: an analysis of the SEER database

**DOI:** 10.1186/s12885-021-08802-8

**Published:** 2021-09-26

**Authors:** Shizhao Cheng, Lei Yang, Xin Dai, Jing Wang, Xingpeng Han

**Affiliations:** 1 Department of Thoracic Surgery, Tianjin Chest Hospital, Tianjin, 300222 China; 2 Department of Thoracic Surgery, Peking University Shenzhen Hospital, Shenzhen, 518035 China; 3 Department of Gastroenterology and Hepatology, Tianjin Medical University General Hospital, Tianjin, 300052 China; 4 Department of Pathology, Tianjin Chest Hospital, Tianjin, 300222 China

**Keywords:** Esophageal cancer, Brain metastases, Risk factor, Prognosis factor, SEER

## Abstract

**Background:**

Brain metastases were rare in esophageal cancer patients. Using the Surveillance, Epidemiology, and End Results (SEER) database, the present study investigated the incidence, risk and prognostic factors of brain metastases in esophageal cancer patients.

**Methods:**

Retrieving esophageal cancer patients diagnosed between 2010 and 2018 from the SEER database, univariable and multivariable logistic and cox regression models were used to investigate the risk factors for brain metastases development and prognosis, respectively. The brain metastases predicting nomogram was constructed, evaluated and validated. The overall survival (OS) of patients with brain metastases was analyzed by Kaplan–Meier method.

**Results:**

A total of 34,107 eligible esophageal cancer patients were included and 618 of them were diagnosed with brain metastases (1.8%). The median survival of the brain metastatic esophageal cancer patients was 5 (95% CI: 5–7) months. The presence of bone metastases and lung metastases were the homogeneously associated factors for the development and prognosis of brain metastases in esophageal cancer patients. Patients younger than 65 years, American Indian/Alaska Native race (vs. White), overlapping lesion (vs. Upper third), esophageal adenocarcinoma histology subtype, higher N stage, and liver metastases were positively associated with brain metastases occurrence. The calibration curve, ROC curve, and C-index exhibited good performance of the nomogram for predicting brain metastases.

**Conclusions:**

Homogeneous and heterogeneous factors were found for the development and prognosis of brain metastases in esophageal cancer patients. The nomogram had good calibration and discrimination for predicting brain metastases.

## Background

Esophageal cancer is the seventh most frequent malignant tumor and the sixth leading cause of death globally, according to the global cancer statistics 2020. Worldwide, an estimated 604,000 new esophageal cancer cases and 544,000 deaths occurred in 2020 [1]. The prognosis of esophageal cancer patients with metastatic disease is dismal [2]. The reported incidence of brain metastases ranged from 0.32 to 13% in esophageal cancer patients [3, 4]. The median survival time of patients with brain metastases was reported to be less than one year [5]. Early diagnosis and intervention in patients with brain metastases could improve survival [6]. However, National Comprehensive Cancer Network (NCCN) and European Society for Medical Oncology (ESMO) guidelines for staging do not recommend performing routine brain imaging in asymptomatic esophageal patients [7]. A predictive nomogram based on the clinicopathologic features of esophageal cancer patients is urgently needed to facilitate metastatic screening. The purpose of the present study was to summarize the incidence, the risk factors, and the prognostic factors of brain metastases in esophageal cancer patients using the Surveillance, Epidemiology, and End Results (SEER) database from 2010 to 2018. Meanwhile, a predictive nomogram was developed and validated to guide the brain metastases screening.

## Methods

### Ethics statement

The present study used previously collected anonymized and de-identified data from the SEER database. Therefore, no additional informed consent was required. The study was complied with the 1964 Helsinki Declaration and its later amendments or comparable ethical standards and deemed exempt from review by the Ethics Board of the Tianjin Chest Hospital.

### Data source

The data used in the present study were abstracted from the SEER 18 registries research database (Nov 2020 Sub, 2000–2018), comprising approximately 30% of the total US population. The information of metastatic sites of brain, bone, liver, and lung were not collected until 2010. So, esophageal cancer patients diagnosed between 2010 and 2018 were included in the present study to analyze brain metastases risk factors. Esophageal cancer patients diagnosed between 2010 and 2017, with a follow-up at least for 1 year, were retrieved to investigate the prognostic factors of esophageal cancer patients with brain metastases. The SEER-stat software (the Surveillance Research Program, National Cancer Institute SEER Stat software, www.seer.cancer.gov/seerstat, Version 8.3.9) was used to generate the case listing.

### Cohort selection

**Fig. 1:**
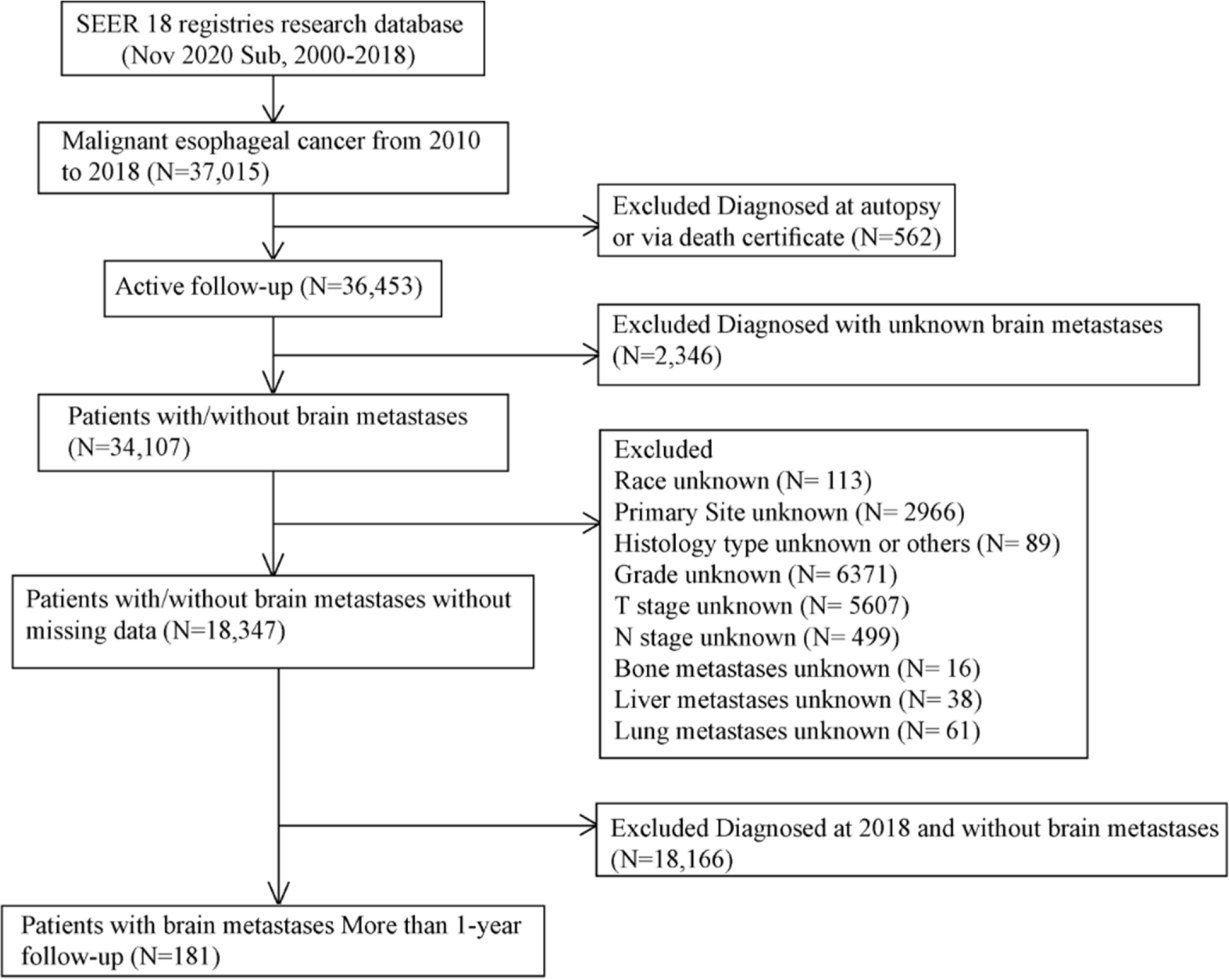
Flowchart of the esophageal cancer patients selection

The inclusion criteria were as follows: the site recodes ICD-O-3 (International Classification of Diseases for Oncology-3)/WHO 2008 was “Esophagus”; the behavior recode for analysis was “Malignant”; diagnosed between 2010 and 2018. The exclusion criteria were as follows: diagnosis obtained from a death certificate or an autopsy; unknown information for brain metastases, or follow-up. The flow-chart for the study population selection was shown in Fig. 1.

### Statistical analysis

Patients’ demographic and clinical features were collected including age, sex, race, primary site, histology type, grade, T stage, N stage, and the presence of bone metastases, liver metastases, lung metastases and survival time. Quantitative data were described as mean ± standard deviation (SD). Categorical data were presented as number and the percentage (N, %). The differences in the brain metastases incidence between the categorical variables were analyzed by Pearson’s chi-square test or rank sum test. The univariable and multivariable logistic regression model were conducted to determine the risk factors of brain metastases. Factors with a *P*-value less than 0.05 in the univariable logistic regression analysis were incorporated into the multivariable regression model.

A nomogram predicting brain metastases was formulated based on the results of multivariable logistic analysis using the rms package in R software (Version 3.4.2; https://www.R-project.org). The performance of the nomogram was evaluated by the receiver operating characteristics (ROC). Calibration curves were plotted to assess the calibration of the nomogram. Harrell’s C-index was measured to quantify the discrimination performance of the nomogram. The nomogram was subjected to bootstrapping validation (1000 bootstrap resamples) to calculate a relatively corrected C-index [8].

The overall survival was analyzed using the Kaplan–Meier method with the log-rank test. The univariable and multivariable Cox regression model were conducted to determine the prognostic factors for patients with brain metastases. Factors with a *P*-value less than 0.05 in the univariable regression analysis were incorporated into the multivariable regression model.

All statistical analyses were performed using the R software (Version 3.4.2; https://www.R-project.org). Two-sided *P* < 0.05 were considered as statistically significant.

## Results

### Patient characteristics

According to the defined inclusion and exclusion criteria, a total of 34,107 patients with esophageal cancer were initially identified. Among them, 26,784 (78.5%) were male, and 7323 (21.5%) were female, mean age was 65.53 ± 11.38 years.

Totally, 618 (1.8%) esophageal cancer patients were initially diagnosed with brain metastases. Among the patients with brain metastases, the majority of cancers (59.1%) were located in the lower third of the esophagus. Esophageal adenocarcinoma (78.2%) was the main histology subtype. More cancers were diagnosed at grade III (42.6%) and N1 (47.6%). 30.3% patients were diagnosed with bone metastases, 37.9% patients with liver metastases and 30.9% patients with lung metastases.

Subsequently, after excluding patients with missing information of race, primary site, histology type, grade, T stage, N stage, bone metastases status, liver metastases status and lung metastases status, we finally included a total of 18,347 patients without missing data in the subsequent univariable and multivariable logistic and cox regression studies and also in the construction of the nomogram predicting brain metastases. The flow-chart for the study population selection was shown in Fig. 1. The demographic and clinical characteristics of the 18,347 esophageal cancer patients without missing data were shown in Table 1.

**Table 1:** Clinical characteristics of 18,347 esophageal cancer patients without missing data, diagnosed from 2010 to 2018

	Without BrM (***N*** = 18,149)	With BrM (***N*** = 198)	***P***-value
**Age (years)**			
< 65	7441 (41.0%)	112 (56.6%)	< 0.001
> =65	10,708 (59.0%)	86 (43.4%)	
**Sex**			
Male	14,305 (78.8%)	174 (87.9%)	0.003
Female	3844 (21.2%)	24 (12.1%)	
**Race**			
White	15,487 (85.3%)	178 (89.9%)	< 0.001
Black	1669 (9.2%)	6 (3.0%)	
Asian or Pacific Islander	878 (4.8%)	9 (4.5%)	
American Indian/Alaska Native	115 (0.6%)	5 (2.5%)	
**Primary site**			
Upper third	1401 (7.7%)	4 (2.0%)	< 0.001
Middle third	3540 (19.5%)	31 (15.7%)	
Lower third	12,450 (68.6%)	144 (72.7%)	
Overlapping lesion	758 (4.2%)	19 (9.6%)	
**Histology**			
Esophageal squamous cell carcinoma	6318 (34.8%)	35 (17.7%)	< 0.001
Esophageal adenocarcinoma	11,831 (65.2%)	163 (82.3%)	
**Grade**			
Grade I	1197 (6.6%)	8 (4.0%)	0.007
Grade II	7886 (43.5%)	67 (33.8%)	
Grade III	8837 (48.7%)	119 (60.1%)	
Grade IV	229 (1.3%)	4 (2.0%)	
**T stage**			
T1	5226 (28.8%)	75 (37.9%)	< 0.001
T2	2438 (13.4%)	19 (9.6%)	
T3	8217 (45.3%)	53 (26.8%)	
T4	2268 (12.5%)	51 (25.8%)	
**N stage**			
N0	7606 (41.9%)	42 (21.2%)	< 0.001
N1	7674 (42.3%)	126 (63.6%)	
N2	2138 (11.8%)	16 (8.1%)	
N3	731 (4.0%)	14 (7.1%)	
**Bone metastases**			
None	17,213 (94.8%)	145 (73.2%)	< 0.001
Yes	936 (5.2%)	53 (26.8%)	
**Liver metastases**			
None	16,365 (90.2%)	134 (67.7%)	< 0.001
Yes	1784 (9.8%)	64 (32.3%)	
**Lung metastases**			
None	17,066 (94.0%)	140 (70.7%)	< 0.001
Yes	1083 (6.0%)	58 (29.3%)	

*BrM* brain metastases

### Risk factors for developing brain metastases

The 18,347 esophageal cancer patients without missing data, diagnosed from 2010 to 2018, were extracted to estimate the risk factors for developing brain metastases. Univariable logistic analysis showed the factors of older age at presentation (OR = 0.53; 95% CI: (0.40–0.71); *P* < 0.001), female patients (OR = 0.51; 95% CI: (0.33–0.77); *P =* 0.002), black race (vs. White; OR = 0.31; 95% CI: (0.12–0.65); *P =* 0.005), T2 stage (vs. T1; OR = 0.54; 95% CI: (0.32–0.88); *P =* 0.018), T3 stage (vs. T1; OR = 0.45; 95% CI: (0.31–0.64); *P* < 0.001) were negatively associated with brain metastases occurrence. However, American Indian/Alaska Native race (vs. White; OR = 3.78; 95% CI: (1.33–8.46); *P =* 0.004), middle third (vs. Upper third; OR = 3.07; 95% CI: (1.21–10.33); *P =* 0.035), lower third (vs. Upper third; OR = 4.05; 95% CI: (1.71–13.19); *P =* 0.006), overlapping lesion (vs. Upper third; OR = 8.78; 95% CI: (3.29–30.37); *P* < 0.001), esophageal adenocarcinoma histology subtype (OR = 2.49; 95% CI: (1.75–3.64); *P* < 0.001), T4 stage (vs. T1; OR = 1.57; 95% CI: (1.09–2.24); *P =* 0.014), N1 stage (vs. N0; OR = 2.97; 95% CI: (2.11–4.27); *P* < 0.001), N3 stage (vs. N0; OR = 3.47; 95% CI: (1.82–6.22); *P* < 0.001), the presence of bone metastases (OR = 6.72; 95% CI: (4.84–9.21); *P* < 0.001), liver metastases (OR = 4.38; 95% CI: (3.22–5.90); *P* < 0.001) and lung metastases (OR = 6.53; 95% CI: (4.75–8.87); *P* < 0.001) were all positively associated with brain metastases risk (Table 2).

**Table 2:** Univariable and multivariable logistic regression for analyzing the risk factors for developing brain metastases in esophageal cancer patients

Subject characteristics	Univariable		Multivariable	
	OR (95% CI)	***P***-value	OR (95% CI)	***P***-value
**Age (years)**				
< 65	1 (reference)		1 (reference)	
> =65	0.53 (0.40–0.71)	< 0.001	0.64 (0.48–0.85)	0.002
**Sex**				
Male	1 (reference)		1 (reference)	
Female	0.51 (0.33–0.77)	0.002	0.74 (0.48–1.16)	0.191
**Race**				
White	1 (reference)		1 (reference)	
Black	0.31 (0.12–0.65)	0.005	0.38 (0.16–0.89)	0.026
Asian or Pacific Islander	0.89 (0.42–1.65)	0.739	1.11 (0.55–2.24)	0.779
American Indian/Alaska Native	3.78 (1.33–8.46)	0.004	3.28 (1.27–8.45)	0.014
**Primary site**				
Upper third	1 (reference)		1 (reference)	
Middle third	3.07 (1.21–10.33)	0.035	2.40 (0.83–6.93)	0.105
Lower third	4.05 (1.71–13.19)	0.006	2.03 (0.71–5.76)	0.186
Overlapping lesion	8.78 (3.29–30.37)	< 0.001	4.02 (1.32–12.25)	0.015
**Histology**				
Esophageal squamous cell carcinoma	1 (reference)		1 (reference)	
Esophageal adenocarcinoma	2.49 (1.75–3.64)	< 0.001	2.01 (1.30–3.10)	0.002
**Grade**				
Grade I	1 (reference)		1 (reference)	
Grade II	1.27 (0.61–2.65)	0.523		
Grade III	2.01 (0.98–4.13)	0.056		
Grade IV	2.61 (0.78–8.75)	0.119		
**T stage**				
T1	1 (reference)		1 (reference)	
T2	0.54 (0.32–0.88)	0.018	0.62 (0.37–1.05)	0.076
T3	0.45 (0.31–0.64)	< 0.001	0.40 (0.28–0.59)	< 0.001
T4	1.57 (1.09–2.24)	0.014	0.96 (0.65–1.41)	0.832
**N stage**				
N0	1 (reference)		1 (reference)	
N1	2.97 (2.11–4.27)	< 0.001	2.66 (1.83–3.85)	< 0.001
N2	1.36 (0.74–2.37)	0.302	1.57 (0.86–2.87)	0.143
N3	3.47 (1.82–6.22)	< 0.001	2.46 (1.29–4.70)	0.007
**Bone metastases**				
None	1 (reference)		1 (reference)	
Yes	6.72 (4.84–9.21)	< 0.001	3.11 (2.19–4.41)	< 0.001
**Liver metastases**				
None	1 (reference)		1 (reference)	
Yes	4.38 (3.22–5.90)	< 0.001	1.51 (1.07–2.14)	0.020
**Lung metastases**				
None	1 (reference)		1 (reference)	
Yes	6.53 (4.75–8.87)	0.001	3.02 (2.11–4.32)	< 0.001

Multivariable analysis further confirmed brain metastases was negatively associated with older age at presentation (OR = 0.64; 95% CI: (0.48–0.85); *P* = 0.002), black race (vs. White; OR = 0.38; 95% CI: (0.16–0.89); *P =* 0.026), T3 stage (vs. T1; OR = 0.40; 95% CI: (0.28–0.59); *P* < 0.001). More brain metastases was positively associated with American Indian/Alaska Native race (vs. White; OR = 3.28; 95% CI: (1.27–8.45); *P =* 0.014), overlapping lesion (vs. Upper third; OR = 4.02; 95% CI: (1.32–12.25); *P* = 0.015), esophageal adenocarcinoma histology subtype (OR = 2.01; 95% CI: (1.30–3.10); *P* = 0.002), N1 stage (vs. N0; OR = 2.66; 95% CI: (1.83–3.85); *P* < 0.001), N3 stage (vs. N0; OR = 2.46; 95% CI: (1.29–4.70); *P* = 0.007), the presence of bone metastases (OR = 3.11; 95% CI: (2.19–4.41); *P* < 0.001), liver metastases (OR = 1.51; 95% CI: (1.07–2.14); *P* = 0.020) and lung metastases (OR = 3.02; 95% CI: (2.11–4.32); *P* < 0.001) (Table 2).

### Performance and validation of the nomogram for predicting brain metastases

**Fig. 2:**
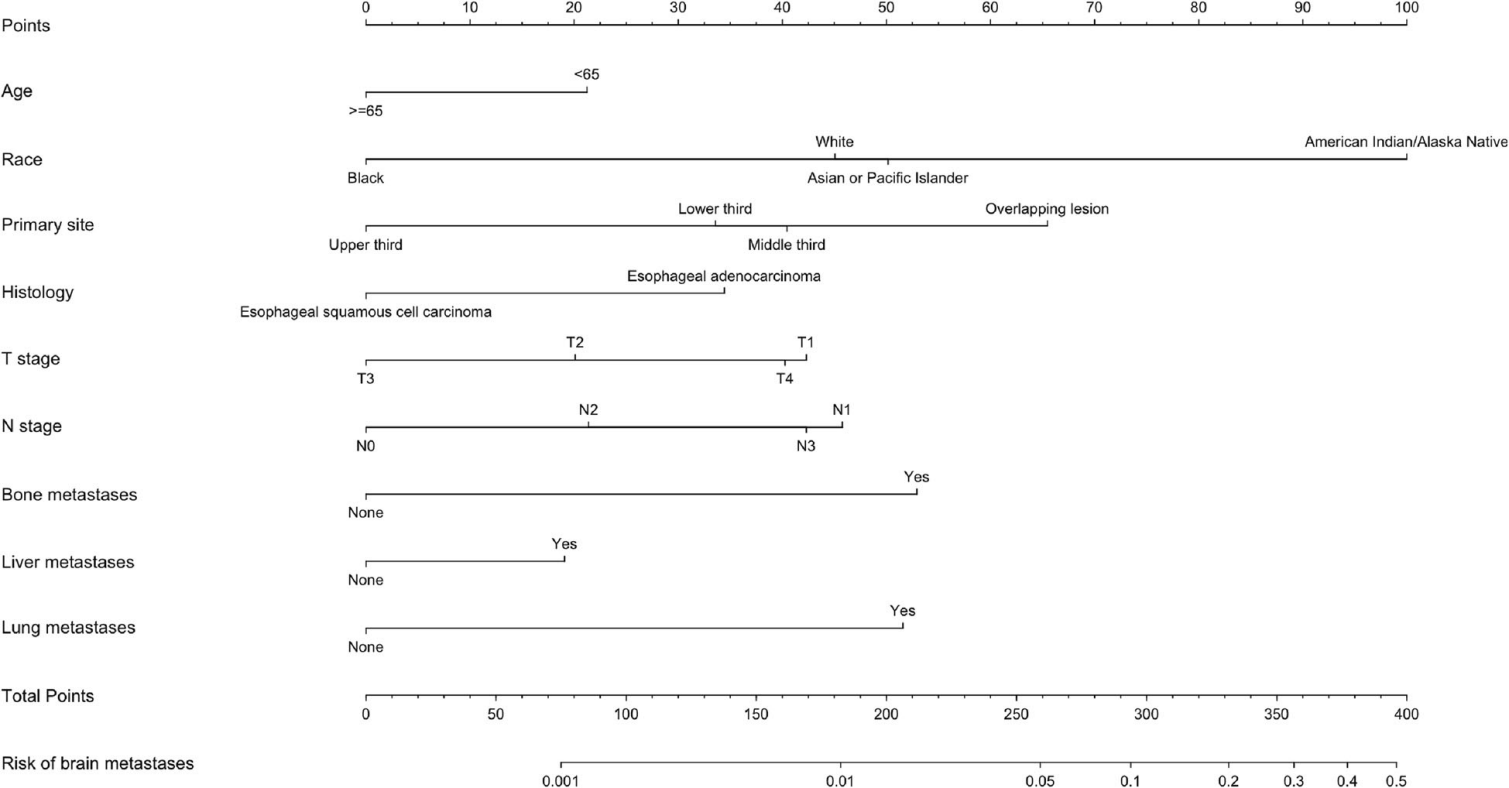
The predicting nomogram for brain metastasis in esophageal cancer patients

**Fig. 3:**
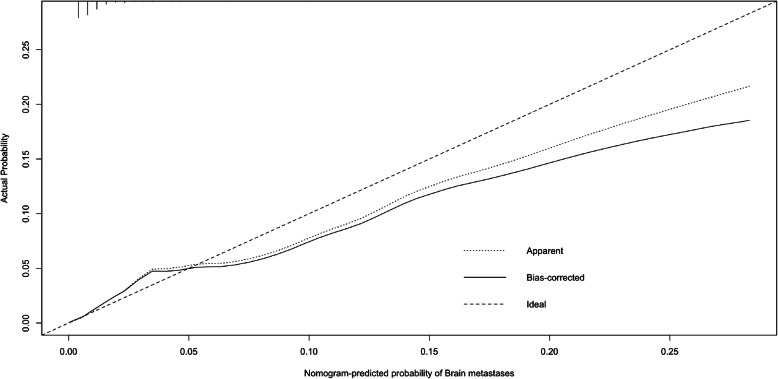
The calibration curves of the predicting nomogram for brain metastasis in esophageal cancer patients

The prediction nomogram that integrated all significant factors for brain metastases in the multivariable logistic regression model was developed and presented in Fig. 2. The calibration curve demonstrated good agreement between the predicted and observed probabilities for brain metastases for esophageal cancer patients (Fig. 3). The Area under the curve (AUC) of the nomogram was 0.817 (95% CI: 0.788–0.846). The C-index for the prediction nomogram was 0.816 (95% CI: 0.796–0.854) and reached 0.801 (95% CI: 0.791–0.845) through bootstrapping validation, which suggested the model’s good discrimination.

### Survival and prognostic factors for esophageal cancer patients with brain metastases

**Fig. 4:**
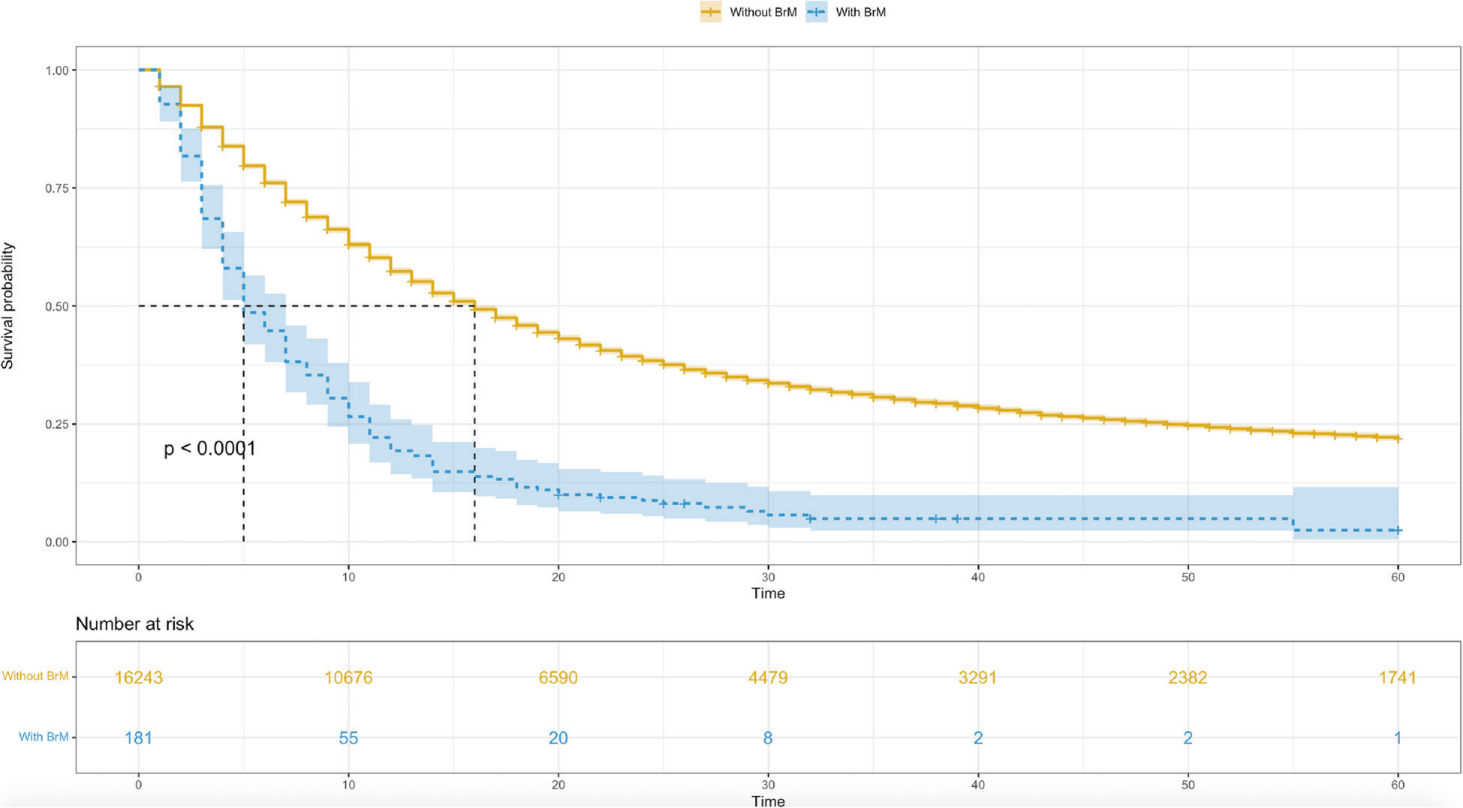
Survival curve for esophagus cancer patients with or without brain metastases. *BrM*: brain metastases

A total of 181 esophageal cancer patients with brain metastases, diagnosed from 2010 to 2017, were extracted to estimate the survival and identify the prognostic factors. The median OS for patients without brain metastases was 16 (95% CI: 16–17) months, and it was decreased to 5 (95% CI: 5–7) months in patients with brain metastases (Fig. 4).

Univariable Cox regression analysis showed improved survival in esophageal adenocarcinoma histology subtype (HR = 0.60; 95% CI: (0.40–0.90); *P* = 0.013), T2 stage (vs. T1; HR = 0.38; 95% CI: (0.21–0.68); *P* = 0.001), T3 stage (vs. T1; HR = 0.63; 95% CI: (0.43–0.94); *P* = 0.023). However, the presence of bone metastases (HR = 2.04; 95% CI: (1.45–2.87); *P* < 0.001), liver metastases (HR = 1.49; 95% CI: (1.08–2.05); *P* = 0.015), and lung metastases (HR = 1.92; 95% CI: (1.38–2.67); *P* < 0.001) showed worse OS (Table 3).

**Table 3:** Univariable and multivariable cox regression for analyzing the prognostic factors for esophageal cancer patients with brain metastases

Subject characteristics	Univariable		Multivariable	
	HR (95% CI)	***P***-value	HR (95% CI)	***P***-value
**Age (years)**				
< 65	1 (reference)		1 (reference)	
> =65	0.90 (0.66–1.22)	0.499		
**Sex**				
Male	1 (reference)		1 (reference)	
Female	1.01 (0.63–1.63)	0.967		
**Race**				
White	1 (reference)		1 (reference)	
Black	1.84 (0.75–4.52)	0.184		
Asian or Pacific Islander	1.63 (0.80–3.32)	0.182		
American Indian/Alaska Native	1.39 (0.51–3.77)	0.516		
**Primary site**				
Upper third	1 (reference)		1 (reference)	
Middle third	1.45 (0.34–6.13)	0.611		
Lower third	1.14 (0.28–4.60)	0.857		
Overlapping lesion	1.69 (0.39–7.32)	0.484		
**Histology**				
Esophageal squamous cell carcinoma	1 (reference)		1 (reference)	
Esophageal adenocarcinoma	0.60 (0.40–0.90)	0.013	0.84 (0.55–1.29)	0.427
**Grade**				
Grade I	1 (reference)		1 (reference)	
Grade II	0.52 (0.25–1.11)	0.09		
Grade III	0.73 (0.36–1.51)	0.398		
Grade IV	0.39 (0.10–1.49)	0.169		
**T stage**				
T1	1 (reference)		1 (reference)	
T2	0.38 (0.21–0.68)	0.001	0.36 (0.20–0.65)	< 0.001
T3	0.63 (0.43–0.94)	0.023	0.66 (0.44–0.99)	0.047
T4	1.16 (0.80–1.68)	0.438	1.21 (0.82–1.78)	0.335
**N stage**				
N0	1 (reference)		1 (reference)	
N1	0.70 (0.48–1.03)	0.073		
N2	0.67 (0.37–1.21)	0.183		
N3	0.90 (0.47–1.70)	0.74		
**Bone metastases**				
None	1 (reference)		1 (reference)	
Yes	2.04 (1.45–2.87)	< 0.001	1.95 (1.34–2.83)	< 0.001
**Liver metastases**				
None	1 (reference)		1 (reference)	
Yes	1.49 (1.08–2.05)	0.015	1.23 (0.87–1.74)	0.239
**Lung metastases**				
None	1 (reference)		1 (reference)	
Yes	1.92 (1.38–2.67)	< 0.001	1.49 (1.03–2.16)	0.035

Multivariable Cox analysis only confirmed T2 stage (vs. T1; HR = 0.36; 95% CI: (0.20–0.65); P < 0.001), T3 stage (vs. T1; HR = 0.66; 95% CI: (0.44–0.99); *P* = 0.047) as the protective factors for patients with brain metastases, while the presence of bone metastases (HR = 1.95; 95% CI: (1.34–2.83); P < 0.001) and lung metastases (HR = 1.49; 95% CI: (1.03–2.16); *P* = 0.035) were risk factors (Table 3).

**Fig. 5:**
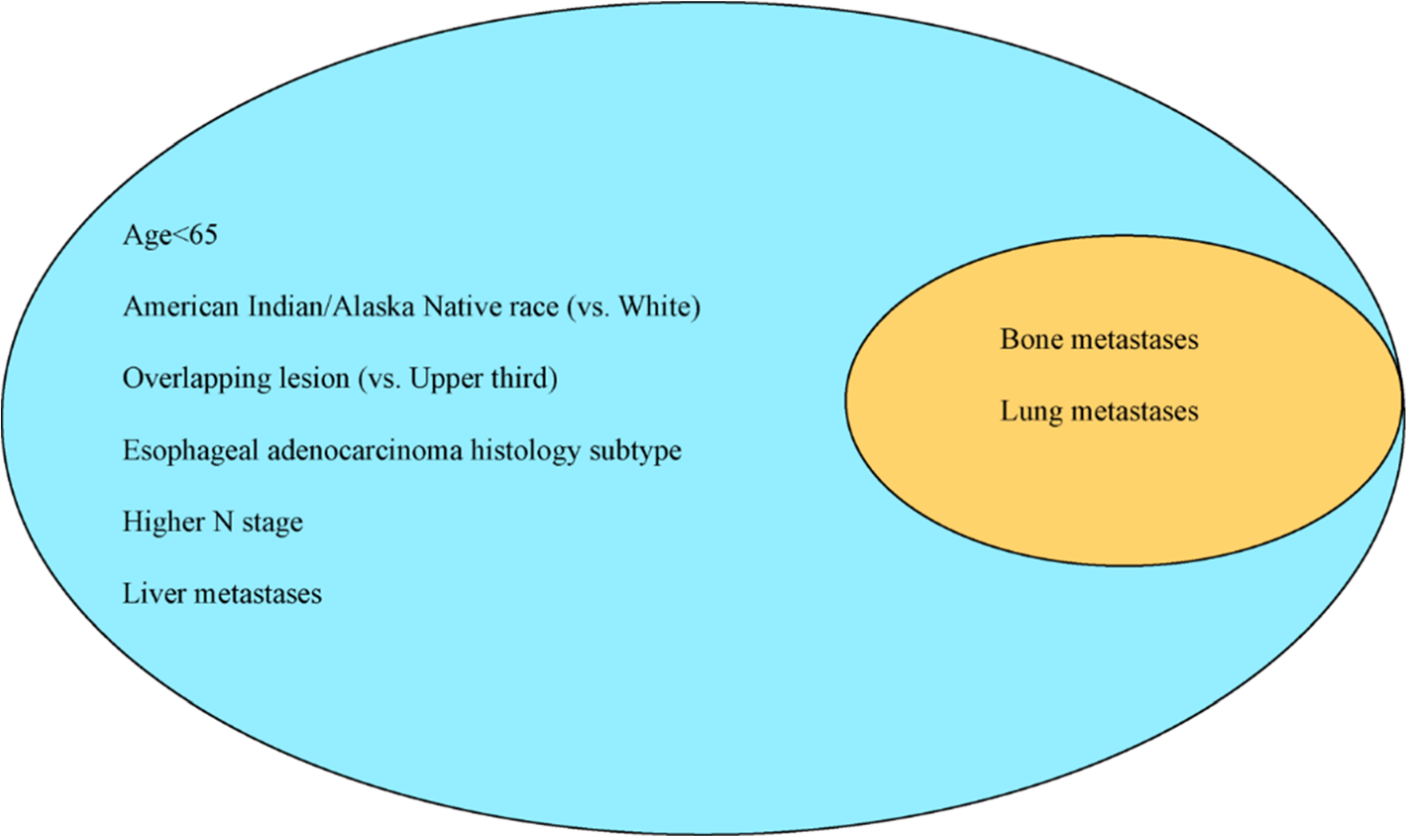
The identification of risk and prognostic factors of brain metastases in esophageal cancer. The factors included in the big circle represented the risk factors for developing brain metastases and the factors in the small circle exhibited homogeneous factors for brain metastases development and prognosis

So, the homogeneous risk factors for the development and prognosis of brain metastases in esophageal cancer were the presence of bone metastases and lung metastases. Patients younger than 65 years, American Indian/Alaska Native race (vs. White), overlapping lesion (vs. Upper third), esophageal adenocarcinoma histology subtype, higher N stage and liver metastases were more likely to have brain metastases occurrence (Fig. 5).

## Discussion

The present study utilized the SEER database to analyze the clinical characteristics, risk factors, and prognostic factors of brain metastases from esophageal cancer. The nomogram to predict brain metastases in esophageal cancer patients was developed and validated.

Due to the low incidence of brain metastases, the available data on brain metastases in esophageal cancer was mainly limited to case reports or single institute studies. The reported incidence of brain metastases ranged from 0.32 to 13% [3, 4]. Two SEER-based studies investigated the patterns of distant metastases in esophageal cancer [2, 9]. The SEER study by Ai and associates included 9934 stage I-IV esophageal cancer patients from 2010 to 2013. Among them, they found 157 patients with brain metastases, the incidence of brain metastases was 1.6% [9]. Another SEER study by Wu and co-workers included 1470 stage IV esophageal cancer patients from 2010 to 2014. Due to the difference in the inclusion and exclusion criteria, only 76 patients with brain metastases were found [2]. Our study included esophageal cancer patients in the SEER database from 2010 to 2018 and found 618 patients with brain metastases. Our results showed the incidence of brain metastases in initial esophageal cancer patients is 1.8%, which was in agreement with previously reported literature.

Next, we used multivariable logistic regression analyses to determine risk factors for brain metastases development. We showed that patients with younger age, American Indian/Alaska Native race (vs. White), overlapping lesion (vs. Upper third), esophageal adenocarcinoma histology subtype, higher N stage, the presence of liver metastases, bone metastases, and lung metastases had an increased risk to develop brain metastases. Partly consistent with our results, a recent SEER study included 28,243 esophageal cancer patients from 2010 to 2016 and analyzed the risk factors for brain metastases. Although T stage and histology grade were not collected from the SEER database, they showed that patients with younger age, overlapping lesion, N1 stage and the presence of extracranial organ metastases were associated with increased risk of brain metastases [10].

Compared with elderly patients, younger patients are more likely to develop brain metastases, which was consistent with the previous studies [9, 11]. Possible reasons for what may have caused differences in brain metastases due to age were investigated by Barz and associates. They found the sclerosis of capillaries of elderly people might be an important factor for the reduced risk of distant organ metastases [11]. In our study, histological subtype was an independent risk factor for brain metastases. Compared with squamous cell carcinoma, the incidence of brain metastases from esophageal adenocarcinoma had doubled. Previous studies reported similar results. After evaluating 157 esophageal cancer patients with brain metastases, Ai and associates found that adenocarcinoma was associated with a higher risk for developing brain metastases, whereas squamous cell cancer was associated with a lower risk of brain metastases [9]. Other studies also reported that the most common histological type accompanied by brain metastases was adenocarcinoma [6, 12]. The mechanism behind the increased risk of brain metastases in esophageal adenocarcinoma may be related to the overexpression of HER2 [13, 14]. However, some previous studies had conflicting results. Welch et al., after a retrospective analysis with 583 esophageal cancer patients from Mayo Clinic, reported no difference in the risk of brain metastases developing between esophageal adenocarcinoma and squamous cell carcinoma [15]. Other studies in Asian countries, including Japan and China, showed a similar incidence of brain metastases equivalent to that in the Western series, although the dominant histology is squamous cell carcinoma [5, 16]. Because of the low incidence of brain metastases, conflicting results between studies may be due to different sample sizes. Nonetheless, the mechanism behind the association between histological subtype and brain metastases occurrence requires further elucidation.

Our study also identified higher N stage and the presence of liver, bone and lung metastases were significantly associated with brain metastases development. These results were in accordance with previous studies. Lin et al. found N stage was an independent risk factor for brain metastases from esophageal squamous cell carcinoma and N0–1 stage was associated with a lower risk of brain metastases (*P* < 0.05) [17]. Wei et al. also showed N1 stage and the presence of extracranial organ metastases were associated with increased risk of brain metastases for esophageal cancer patients [10]. Overall, lymph node metastases and extracranial metastases represented high tumor burden, thus increasing the risk of brain metastases.

Also, we developed and validated a nomogram to predict brain metastases in esophageal cancer patients using the statistically significant variables in the multivariate logistic analysis. Internal validation showed good discrimination and calibration power. Physicians could use the nomogram as a tool to perform metastatic screening for esophageal cancer patients with a high brain metastases risk.

It should be also noted that, as shown in the predictive nomogram, the T1 stage and N1 stage were shown as stronger predictive factors for brain metastases than the more advanced stage. Indeed, a recent case study reported brain metastases from a T1N1 asymptomatic esophageal adenocarcinoma patient [18]. The brain lesions of the patient were first discovered and removed, and the pathological examination of the brain lesions considered gastrointestinal tract tumor metastases. During the following whole-body examination, the asymptomatic esophageal cancer was found and esophagus resection was performed. Pathological diagnosis confirmed brain metastases from the esophageal cancer. The final pathological stage was T1N1M1, stage IV. Therefore, a distinct mechanism behind brain metastases of esophageal cancer may be due to a direct metastatic pathway through the vertebral venous system to the brain, as described for Batson’s plexus [19].

In our study, the median OS for patients with brain metastases was 5 months and the presence of bone metastases and lung metastases were associated with poor prognosis. Consistent with our results, a systematic review by Ghidini and associates included twenty-one studies from 1991 to 2016 to investigate the clinical outcome and molecular characterization of brain metastases from esophageal cancer. They found the median OS from diagnosis of brain metastases was 4.2 months. They concluded performance status, multimodal therapy, adjuvant chemotherapy, single brain lesion, isolated brain metastases and surgery were prognostic factors for OS [20].

However, the present study has several limitations. First, the SEER database merely recorded the presence/absence of brain metastases based on the initial diagnosis. Symptoms and diagnosis methods for brain metastases were not reported. Second, subsequent treatment of brain metastases and the response after treatment were not available in the public SEER database. Third, the information on performance status, treatment modality, and tumor marker data were not collected in the SEER database, which may reduce the prediction value of the nomogram. Fourth, the variables in the SEER database contained numerous missing values, but through strict inclusion and exclusion criteria to exclude the patients with missing values, the statistical analysis of the subsequent univariable and multivariable logistic and cox regression was scientifically acceptable and reliable.

## Conclusions

The present SEER study provided insight into the epidemiology of brain metastases in newly diagnosed esophageal cancer patients. The prevalence of brain metastases in esophageal cancer patients was 1.8%. The median overall survival time of brain metastases was 5 months. Results showed homogeneous and heterogeneous associated factors for brain metastases development and prognosis. The nomogram had good performance for predicting brain metastases development. Imaging examination of the central nervous system should be considered for esophageal cancer patients with a high brain metastases risk.

## Data Availability

The data were abstracted from the Surveillance, Epidemiology, and End Results (SEER) database. This is an open database. (https://seer.cancer.gov).

## Abbreviations

SEER: Surveillance, Epidemiology, and End Results database
OS: Overall survival
AUC: Area under the curve
NCCN: National Comprehensive Cancer Network
ESMO: European Society for Medical Oncology
BrM: Brain metastases.
